# Adherence to Drug Label Recommendations for Avoiding Drug Interactions Causing Statin-Induced Myopathy–A Nationwide Register Study

**DOI:** 10.1371/journal.pone.0069545

**Published:** 2013-08-06

**Authors:** Jennifer Settergren, Birgit Eiermann, Buster Mannheimer

**Affiliations:** 1 Karolinska Institutet Department of Clinical Science and Education at Södersjukhuset, Stockholm, Sweden; 2 Karolinska Institutet Department of Laboratory Medicine, Division of Clinical Pharmacology, Karolinska University Hospital, Huddinge, Sweden; Sapienza University of Rome, Italy

## Abstract

**Purpose:**

To investigate the extent to which clinicians avoid well-established drug-drug interactions that cause statin-induced myopathy. We hypothesised that clinicians would avoid combining erythromycin or verapamil/diltiazem respectively with atorvastatin or simvastatin. In patients with statin-fibrate combination therapy, we hypothesised that gemfibrozil was avoided to the preference of bezafibrate or fenofibrate. When combined with verapamil/diltiazem or fibrates, we hypothesized that the dispensed doses of atorvastatin/simvastatin would be decreased.

**Methods:**

Cross-sectional analysis of nationwide dispensing data. Odds ratios of interacting erythromycin, verapamil/diltiazem versus respective prevalence of comparator drugs doxycycline, amlodipine/felodipine in patients co-dispensed interacting statins simvastatin/atorvastatin versus patients unexposed (pravastatin/fluvastatin/rosuvastatin) was calculated. For fibrates, OR of gemfibrozil versus fenofibrate/bezafibrate in patients co-dispensed any statin was assessed.

**Results:**

OR of interacting erythromycin versus comparator doxycycline did not differ between patients on interacting and comparator statins either in patients dispensed high or low statin doses (adjusted OR 0.87; 95% CI 0.60–1.25 and 0.92; 95% CI 0.69–1.23). Interacting statins were less common among patients dispensed verapamil/diltiazem as compared to patients on amlodipine/felodipine (OR high dose 0.62; CI 0.56–0.68 and low dose 0.63; CI 0.58–0.68). Patients on any statin were to a lesser extent dispensed gemfibrozil compared to patients not dispensed a statin (OR high dose 0.65; CI 0.55–0.76 and low dose 0.70; CI 0.63–0.78). Mean DDD (SD) for any statin was substantially higher in patients co-dispensed gemfibrozil 178 (149) compared to patients on statin monotherapy 127 (93), (p<0.001).

**Conclusions:**

Prescribers may to some extent avoid co-prescription of statins with calcium blockers and fibrates with an increased risk of myopathy. We found no evidence for avoiding co-prescriptions of statins and antibiotics with an increased risk of statin-induced adverse drug reactions. Co-prescription of statins and gemfibrozil is paradoxically associated with a marked increased statin dose, further aggravating the risk for severe myopathy.

## Introduction

The introduction of HMG-CoA reductase inhibitors, (statins) has had a major impact on the modern management of cardiovascular disease. In high risk populations, statin treatment decreases morbidity and mortality by 30% and has therefore become widely used [1].

Although considered relatively safe, statins are associated with adverse drug reactions [2] including statin induced myopathy. The incidence of rhabdomyolysis has, in clinical trials, ranged from 0.1–0.6% [3] which may underestimate the occurrence in ‘real world’ patients due to the exclusion of patients with risk factors. Known risk factors are increased age, female gender, dispensed statin dose and combination of drugs that increase the statin plasma level including erythromycin, diltiazem, verapamil and gemfibrozil [4]–[9]. There are currently five statins available on the Swedish market: simvastatin, atorvastatin, pravastatin, fluvastatin and rosuvastatin. The metabolism of simvastatin and atorvastatin depends heavily on the hepatic cytochrome P450 enzyme (CYP) 3A4 and is therefore susceptible for interactions with macrolides such as erythromycin, the calcium antagonists diltiazem and verapamil, – potent inhibitors of this enzyme [2], [10]–[18]. The remaining statins, are either eliminated unchanged (pravastatin and rosuvastatin) or are subject for a clearance based on other metabolic pathways (fluvastatin), and may therefore be an alternative for patients in need of long-term treatment with these drugs [2], [11], [14], [19]–[21]. Another strategy may be to use drugs with a similar indication that do not significantly inhibit CYP3A4 such as doxycycline instead of erythromycin and any of the alternative calcium antagonists amlodipine or felodipine instead of diltiazem or verapamil, or to decrease the administered statin dose [22]–[24]. Fibrates are another group of drugs used for the treatment of patients with hyperlipidemia. Gemfibrozil increases the plasma level for most statins due to interaction on transport protein level and has been associated with a large increased risk for myopathy (OATPBA1, p-glycoprotein P) [3], [5], [25]–[30]. For the group of patients at risk for stroke or heart infarction already being treated with a statin and in need for additional lipid-lowering treatment, a better alternative would therefore be fenofibrate or bezafibrate (Table 1) [5], [27], [31]–[34]. These differences are also reflected by respective labelling of the drugs and can be recognised using an interaction software program. In Sweden, SFINX (Swedish Finnish interaction X referencing) alerts approximately 80% of Swedish doctors for drug-drug interactions at the moment of prescribing [35]–[37].

**Table 1:** Rationale for the choice of study drugs.

Labelled indications for groups of drugs according to FASS^1^	Drugs (ATC code^2^)	Role of study drug	Rationale	Labelling according to FASS^1^ with regard to interactions leading to myopathy
**HMG-CoA** 3	Simvastatin	CYP3A4^4^	Metabolism heavily dependent on	Co-administration with erythromycin is
**Reductase inhibitors**	(C10AA01)	interacting	CYP3A4^4^. Its combination with	contraindicated. Co-administration with
**statins**		statin	inhibitors of this enzyme results in a	verapamil and gemfibrozil should be
Used as first line			marked increase in plasma levels	avoided. Co-administration with diltiazem
treatment against			11,12,14] and an elevated risk of	and verapamil should be used with caution
hyper			myopathy [2].	and include close clinical monitoring /
cholesterolemia				dose reductions.
	Atorvastatin	CYP3A4^4^	Metabolism dependent on	Combination with inhibitors of CYP3A4^4^
	(C10AA05)	interacting	CYP3A4^4^ [10]. Its combination with	such as erythromycin, diltiazem and
		statin	inhibitors of this enzyme results in an	verapamil should be avoided/include
			increase in plasma level [14], [16], [17]	close clinical monitoring/dose reductions.
			and an elevated risk of myopathy	
			2,15].	
	Pravastatin	Comparator	Comparator statin. Mainly excreted	No warnings are issued regarding the co-
	(C10AA03)	statin	unchanged through the kidneys and	administration with inhibitors of
			via the bile into the faeces and not	CYP3A4^4^.
			subject for significant interactions on	
			the basis of CYP3A4^4^ inhibition	
			11,14,19].	
	Fluvastatin	Comparator	Is extensively metabolized by CYP2C9^5^	No warnings are issued regarding the co-
	(C10AA04)	statin	and not subject for significant interactions	administration with inhibitors of CYP3A4^4^.
			on the basis of CYP3A4^4^ inhibition [20].	
	Rosuvastatin	Comparator	Comparator statin. Ninety percent is being	No warnings are issued regarding the co-
	(C10AA07)	statin	excreted unchanged through the faeces [2].	administration with inhibitors of CYP3A4^4^.
			Plasma levels not elevated in the presence	
			of potent inhibitors of CYP3A4^4^ [21].	
**Antibiotics**	Erythromycin	Interacting	A macrolide being a potent inhibitor of	Should not be used concomitantly with
Used against airway	(J01FA01)	antibiotic	CYP3A4^4^ and p-glykoprotein [12], [16].	simvastatin or atorvastatin.
infections, atypical	Doxycycline	Comparator	A tetracycline being metabolized to a very	No warnings are issued regarding the co-
pneumonia and	(J01AA02)	antibiotic	little extent [22].	administration with statins.
patients allergic to				
penicillin.				
Calcium -	Verapamil	Interacting	Strong inhibitor of CYP3A4^4^. Has	Co-administration with simvastatin, or
antagonists	(C08DA01)	calcium	increased simvastatin exposure and been	atorvastatin, should be used with caution and
Sharing indications		antagonist	associated with rhabdomyolysis [2], [12].	lead to a dose reduction.
against hypertension	Diltiazem	Interacting	Inhibitor of CYP3A4^4^. Increased	Co-administration with a statin metabolised
and angina pectoris.	(C08DB01)	calcium	simvastatin exposure and is associated	by CYP3A4^4^ should be avoided or include
Only verapamil and		antagonist	with rhabdomyolysis [18].	close clinical monitoring.
diltiazem may be used	Amlodipine	Comparator	Weak inhibitor CYP3A4^4^. Clinically	No warnings are issued regarding the co-
**for the treatment of**	(C08CA01)	calcium	significant interactions with statins are	administration with statins.
atrial fibrillation.		antagonist	unlikely [23], [24].	No warnings are issued regarding the co-
	Felodipine	Comparator	No significant inhibitor of CYP3A4^4^ [24].	administration with statins.
	(C08CA02)	calcium		
		antagonist		
**Fibrates**	Gemfibrozil	Interacting	Marked and clinically significant	Co-administration with statins should be
Used for the treatment	(C10AB04)	fibrate	pharmacokinetic interactions with statins	avoided.
of			on the basis of membrane transporter	
hypertriglyceridemia			inhibition [25]–[30]. Concomitant use is	
or when treatment of			associated with an increased risk of	
statin is not tolerated			rhabdomyolysis [2], [3], [5].	
	Fenofibrate	Comparator	Do not affect the plasma exposure of	Due to a pharmacodynamic interaction co-
	(C10AB05)	fibrate	statins [31]–[34].	administration with statins should be used
				with caution.
	Bezafibrate	Comparator	Do not affect the plasma exposure of	Due to a pharmacodynamic interaction co-
	(C10AB02)	fibrate	statins [27].	administration with statins should be used
				with caution.

1 The Swedish summary of product characteristics [35].

2 Anatomical Therapeutic Chemical code.

3 3-hydroxy-3-methylglutaryl-coenzyme A.

4 Cytochrome P450 enzyme (CYP) 3A4.

5 Cytochrome P450 enzyme (CYP) 2C9.

The current study aims to investigate the compliance to guidelines on drug-drug interactions with the potential to cause statin induced myopathy on behalf of the prescribing doctors. We hypothesised that physicians in Sweden would avoid the combined use of statins and drugs that inhibit their metabolism and/or, when combined with drugs used on continuous basis, i.e. verapamil/diltiazem or fibrates, reduce the statin dose.

## Methods

### Ethics statement

This was a database study that included data on the entire Swedish population 18 years or older. Hence we did not interfere with the treatment of these individuals nor in any other way. Since the data was anonymized and none of the individuals were identifiable, the integrity of the individuals was not judged to be violated. This view was also supported by the Regional Ethics Committee in Stockholm, Karolinska Institute, which waived the need for written informed consent from the participants and approved the study as a whole.

### Study design

The study design was a retrospective, cross-sectional analysis of patients being dispensed prescription drugs in Sweden during the period from 15 August to 15 December 2011. The choice of a four-month-study-period was based on the Swedish regulation and experience that most patients on long-term/chronic treatment repeat their drug-dispensing every third to fourth month. We selected all individuals, 18 years or older, that were dispensed any of the drugs presented in Table 1. The cohort was established on data obtained from the Swedish Prescribed Drug Register [38].

### Data source

The Swedish Prescribed Drug Register contains data with unique patient identifiers for all dispensed prescriptions for the whole population of Sweden. The data collection is administered by the National Corporation of Swedish Pharmacies, a state-owned company responsible for the provision of pharmaceutical services at a nationwide level. Data on all dispensed prescriptions is transferred monthly to the National Board of Health and Welfare. The drugs were classified according to the Anatomical Therapeutic Chemical (ATC) classification system. We selected all individuals, 18 years and older, dispensed any of the drugs presented in Table 2.

**Table 2:** Prevalence of study drugs used in the adult Swedish population (≥18 years of age) and corresponding demographics, 15^th^ August to 15^th^ December, 2011.

Study drugs	n	n/1000 individuals	Mean age (SD^1^)	Women (%)	Percentage of drugs^2^ prescribed from primary care	Mean number of drugs (SD^1^)	Mean dispensed DDD^3^ statin (SD^1^)
**CYP3A4** 4							
**interacting**							
**statins**							
simvastatin	562 723	74	69 (11)	45	84 (459075/548 863)	6.8 (3.9)	113 (68)
atorvastatin	75 950	10	67 (10)	43	70 (51 773/73 968)	7.6 (4.2)	209 (159)
**Comparator**							
**statins**							
rosuvastatin	26 964	3.6	65 (10)	44	67 (17 417/25 939)	7.2 (4.2)	202 (155)
pravastatin	14 891	2.0	70 (11)	50	83 (12 056/14 608)	7.6 (4.3)	125 (66)
fluvastatin	311	0.041	71 (11)	61	78 (163/209)	7.6 (4.6)	67 (47)
**CYP3A4** 3							
**interaction with**							
**statins**							
erythromycin^5^	8 795	1.2	46 (17)	66	74 (6 286/8 505)	5.4 (4.6)	NA^6^
verapamil	14 114	1.9	72 (13)	61	76 (10 522/13820)	7.7 (4.4)	NA
diltiazem	11 341	1.5	73 (11)	58	79 (8 726/11 091)	8.7 (4.5)	NA
**Pharmacokinetic**							
**interaction with**							
**all statins**							
gemfibrozil	3 726	0.49	66 (11)	33	84 (3 055/3 638)	7.9 (4.5)	NA
**Comparator**							
**drugs**							
doxycycline	143 564	19	52 (18)	59	81 (112 531/138 641)	5.9 (4.6)	NA
amlodipine	255 969	34	69 (12)	47	86 (216 892/251 344)	6.6 (3.9)	NA
felodipine	209 425	27	71 (12)	50	87 (177 688/204 341)	6.8 (3.9)	NA
bezafibrate	2 571	0.34	66 (11)	41	80 (2 017/2 509)	8.5 (4.5)	NA
fenofibrate	1 728	0.23	63 (11)	38	67 (1 126/1 687)	8.1 (4.7)	NA

1 Standard Deviation.

2 Defined as a seven-digit Anatomical Therapeutic Chemical (ATC) code. The remaining proportions were prescribed from a specialist care setting.

3 Mean dispensed Daily Defined Dose.

4 Cytochrome P450 enzyme (CYP) 3A4.

5 In addition to affect CYP3A4 erythromycin is a potent inhibitor of p-glycoprotein.

6 Non Applicable.

### Variables

We hypothesised that physicians in Sweden would avoid the combined use of statins and drugs that inhibit their metabolism. If so, the odds ratio between the prevalences of interacting drugs to comparator drug users would be lower among patients co-dispensed a statin whose metabolism may be inhibited, similar to a methodology used previously [39]:



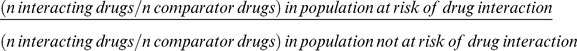



Thus, for antibiotics and calcium antagonists, the outcome measures were odds ratios of each of the two types of interacting drugs (erythromycin, verapamil/diltiazem) versus respective comparator drugs (doxycycline, amlodipine/felodipine) in patients co-dispensed interacting statins (simvastatin, atorvastatin) versus patients being unexposed (instead dispensed pravastatin, fluvastatin, rosuvastatin). For fibrates, the outcome measure was odds ratios of gemfibrozil versus fenofibrate/bezafibrate in patients co-dispensed any type of statins. To investigate the effect of statin dose on prescribing patterns, respective outcome variable was split into 3 categories where odds ratios in patients dispensed high and low daily dose statins were compared with a respective reference population. Daily doses for respective drug were estimated by dividing the total amount dispensed daily defined doses (mg) with the number of days included in the study period (n = 122) [40]. High statin daily doses were defined as ≥40 mg for atorvastatin, fluvastatin, pravastatin, and simvastatin and ≥20 mg for rosuvastatin.

In the statistical analysis, factors considered potential effect modifiers were age, gender and medical setting. The variable age was divided into three groups (18–45, 46–64 and ≥65) and, along with the other explanatory variables treated as category variables. Information on medical setting was based on the variable “Prescribers’ working place” in the SPDR and whether the lipid lowering drug was being prescribed from a primary or specialist care unit. Primary care was defined as care provided by health care professionals that often play a role in the local community and act as a first point of consultation for all patients within the health care system. Secondary care was defined as care provided by medical specialists often associated with a hospital such as cardiologists, endocrinologists or other internists.

Regarding the treatment groups airway antibiotics and calcium antagonists, the medical setting was defined according to the place where respective statins were dispensed. In order to be able to categorize the reference group for the fibrates, the medical setting was instead defined from the place which the respective fibrate was dispensed.

In addition to these odds ratios, dispensed Defined Daily Doses (DDD) was estimated for the Swedish population as a whole and in specific subgroups (mean, SD) [40]. Concomitantly dispensed drugs were defined as the occurrence of several unique 7-digit ATC codes within the study period.

### Analysis

To study associations between statins and the interacting drugs and to control for potential effect modifiers we used unconditional logistic regression. The associations are presented as odds and odds ratios (OR) with 95% confidence intervals (CI). The departure from 1 (no association) is statistically significant at the 5% level, two-tailed, if the 95% CI does not include 1. All statistical calculations were performed in IBM SPSS Statistics 20.0 (SPSS Inc., Chicago, IL, USA). Two sample t-tests were used to compare different means.

## Results

**Figure 1:**
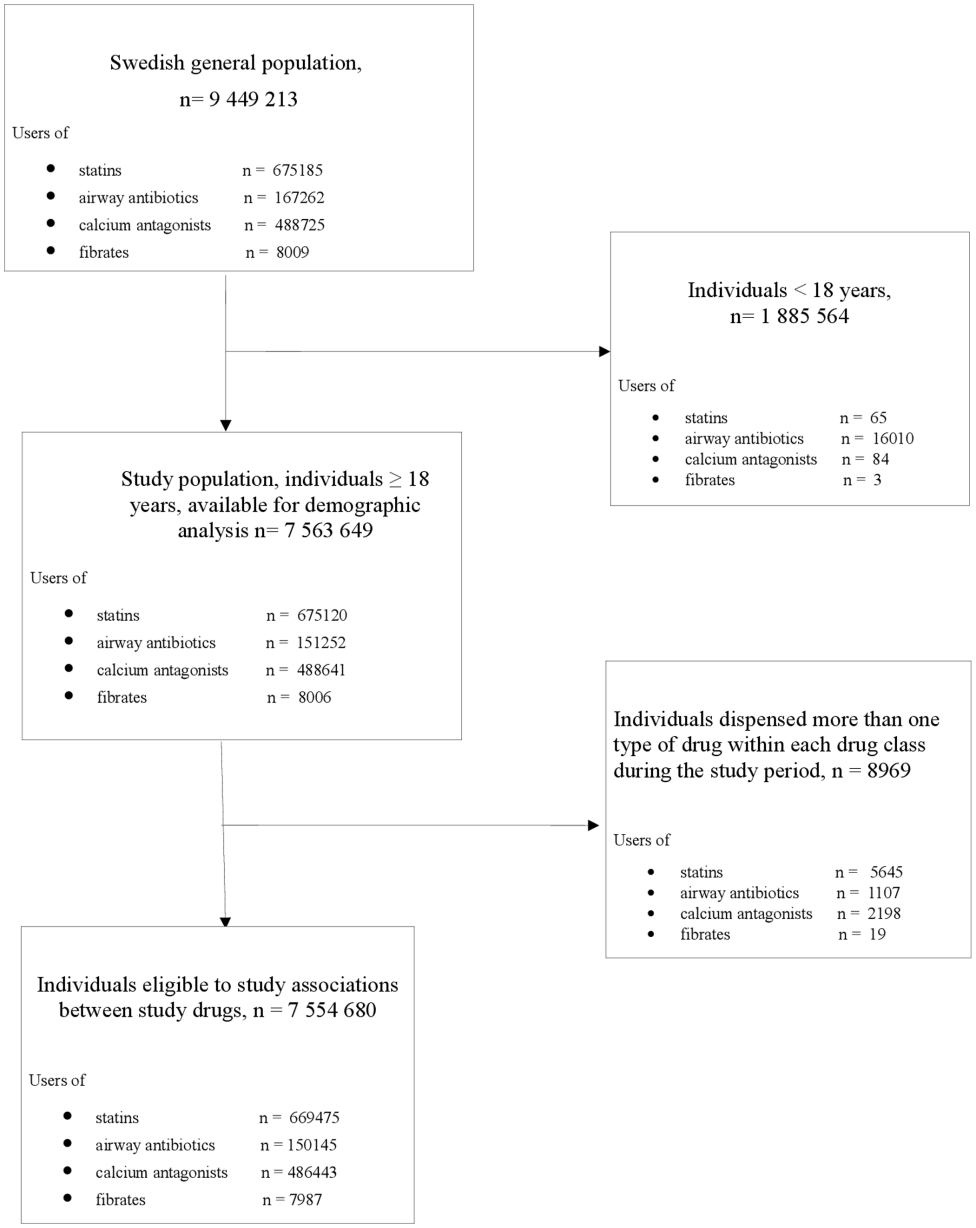
Patient flow chart.

Individuals in the Swedish population 18 years and older (n = 7 563 649) were included in the study [41]. To minimize the possible bias of patients who changed interacting drugs, comparator drugs and/or statins within the 4-month-study period, associations between different classes of drugs was based on the 7 554 680 individuals who had been dispensed no more than one of the drugs in each therapeutic area (e.g. those who had been dispensed both an interacting drug and a comparator drug were excluded) (Figure 1).

The mean age was 49 years, and 51% were women. The prevalence of the use of study drugs in the Swedish study population is given in Table 2 along with corresponding demographics. Nine percent of the population was dispensed any kind of statin where simvastatin was the most common (7%) (Table 2).

Table 3 shows the prevalences of statins dispensed in combination with potentially interacting drugs in patients dispensed high and low doses of statins. The number of patients dispensed simvastatin/atorvastatin in combination with erythromycin and verapamil/diltiazem was in all 520 and 8445 respectively. The number of patients dispensed any statin in combination with gemfibrozil was 1292.

**Table 3:** Associations between statins, in patients dispensed drugs with or without a pronounced inhibitory effect on their metabolism.

Statins	Groups ofdispensedstatin dose^1^	Interactivedrugs n (%)	Comparator drugsn (%)	OddsInteractive drugs(vs. Comparatordrugs)	Odds ratiosUnadjusted(95% CI^2^)	p-values	Odds ratios -adjusted forage, gender, andmedical setting(95% CI^2^)	p-values
		**erythromycin**	**doxycycline**					
		**(n = 576)**	**(n = 16199)**					
**CYP3A4** 3	High dose	76 (13)	2157 (13)	0.035	0.85 (0.60–1.20)	0.35	0.87 (0.60–1.25)	0.44
**interacting statins:**								
**simvastatin/**								
**atorvastatin**								
	Low dose	444 (77)	12697 (78)	0.035	0.84 (0.63–1.12)	0.23	0.92 (0.69–1.23)	0.56
**Comparator**		56 (10)	1345 (8)	0.042				
**statins: pravastatin/**								
**fluvastatin/**								
**rosuvastatin**								
**(reference group)**								
		**verapamil/**	**amlodipine/**					
		**diltiazem**	**felodipine**					
		**(n = 9246)**	**(n = 176168)**					
**CYP3A4** 3 **interacting**	High dose	1183 (13)	24130 (14)	0.046	0.59 (0.54–0.65)	<0.001	0.62 (0.56–0.68)	<0.001
**statins: simvastatin/**								
**atorvastatin**								
	Low dose	7262 (79)	142334 (81)	0.051	0.62 (0.57–0.67)	<0.001	0.63 (0.58–0.68)	<0.001
**Comparator statins:**		801 (9)	9704 (6)	0.083				
**pravastatin /**								
**fluvastatin /**								
**rosuvastatin**								
**(reference group)**								
		**gemfibrozil**	**fenofibrate/**					
		**(n = 3712)**	**bezafibrate**					
			**(n = 4275)**					
**Pharmacokinetic**	High dose	326 (9)	514 (12)	0.63	0.63 (0.54–0.73)	<0.001	0.65 (0.55–0.76)	<0.001
**interaction with**								
**gemfibrozil: any statin**								
	Low dose	966 (26)	1351 (32)	0.72	0.71 (0.64–0.79)	<0.001	0.70 (0.63–0.78)	<0.001
**no statin**		2420 (65)	2410 (56)	1.00				
**(reference group)**								

Data are based on drug dispensing in individuals ≥18 years of age (n = 7 554 680), 15 August to 15 December, 2011.

1 High statin doses were defined as 40 mg or more for atorvastatin, fluvastatin, pravastatin.

and simvastatin and 20 mg or more for rosuvastatin.

2 Confidence Intervals.

3 Cytochrome P450 enzyme (CYP) 3A4.

The associations between interacting drugs and comparator drugs in the population as a whole and in subgroups were consistently investigated in individuals with high and low statin doses. The odds ratio of the interacting erythromycin versus the comparator doxycycline did not differ between patients on interacting and comparator statins either in patients dispensed high or low doses (adjusted OR 0.87; 95% CI 0.60–1.25 and 0.92; 95% CI 0.69–1.23). However, interacting statins were significantly less common among patients dispensed verapamil/diltiazem as compared to patients on comparator drugs amlodipine/felodipine (adjusted OR 0.62; CI 0.56–0.68 and 0.63; CI 0.58–0.68). Similarly, patients on any kind of statin were less likely to be dispensed gemfibrozil as compared to patients not dispensed a statin (adjusted OR 0.65; CI 0.55–0.76 and 0.70; CI 0.63–0.78).

Table 4 shows the adjusted odds ratios between statins in patientś dispensed drugs with or without a pronounced inhibitory effect on their metabolism in primary care settings, specialist care settings, individuals ≥65 years of age, and in females. The results in the investigated subgroups were mostly consistent with those for the general population. However some differences were noted. The decreased association between interacting statins and patients with verapamil/diltiazem was markedly more pronounced in patients treated in specialist care settings (adjusted OR high dose and low dose statins 0.39; CI 0.33–0.46 and 0.39; CI 0.34–0.44) as compared to patients treated in a primary care settings (adjusted OR 0.79; CI 0.70–0.89 and 0.80; CI 0.72–0.88). In females interacting gemfibrozil versus the comparator fenofibrate/bezafibrate did not differ significantly between individuals on any kind of statin and the females that were not dispensed a statin (adjusted OR 0.92; CI 0.68–1.24 and 0.90; CI 0.75–1.08). Among patients 65 years or older, a trend, however not significant, towards a decreased association between interacting statins and patients with erythromycin was noted (adjusted OR 0.70; CI 0.42–1.17 and 0.74; CI 0.50–1.10).

**Table 4:** Associations between statins, in patientś dispensed drugs with or without a pronounced inhibitory effect on their metabolism in primary care setting, specialist care setting, elderly individuals ≥65 years of age and in females under the study period 15 August to 15 December, 2011.

Statins	Groups ofdispensedstatindoses^1^	Individualsprescribed fromprimary care,adjusted^2^ odds ratios(95% CI^3^)	Individualsprescribed fromspecialised care,adjusted^2^ odds ratios(95% CI^3^)	Individuals ≥65years(n = 1 779 439),adjusted^4^ oddsratios (95% CI^3^)	Females (n = 3 818 524),adjusted^5^ odds ratios(95% CI^3^)
**CYP3A4** 6	High dose	1.01 (0.64–1.60)	0.84 (0.53–1.35)	0.70 (0.42–1.17)	0.88 (0.52–1.49)
**interacting statins:**					
**simvastatin/**					
**atorvastatin**					
	Low dose	0.97 (0.67 – 1.42)	0.65 (0.35–1.19)	0.74 (0.50–1.10)	1.13 (0.75–1.70)
**Comparator statins:**					
**pravastatin/**					
**fluvastatin/**					
**rosuvastatin**					
**(reference group)**					
**CYP3A4** 6	High dose	0.79 (0.70–0.89)	0.39 (0.33–0.46)	0.71 (0.63–0.79)	0.66 (0.58–0.76)
**interacting statins:**					
**simvastatin/**					
**atorvastatin**					
	Low dose	0.80 (0.72–0.88)	0.39 (0.34–0.44)	0.74 (0.67–0.81)	0.69 (0.62–0.77)
**Comparator statins:**					
**pravastatin/**					
**fluvastatin/**					
**rosuvastatin**					
**(reference group)**					
**Pharmacokinetic**	Highdose	0.65 (0.54–0.77)	0.65 (0.48–0.88)	0.61 (0.49–0.77)	0.92 (0.68–1.24)
**interaction with**					
**gemfibrozil: any**					
**statin**					
	Low dose	0.71 (0.63–0.80)	0.68 (0.54–0.86)	0.75 (0.65–0.86)	0.90 (0.75–1.08)
**no statin**					
**(reference group)**					

1 High statin doses were defined as 40 mg or more for atorvastatin, fluvastatin, pravastatin and simvastatin and 20 mg or more for rosuvastatin.

2 Estimates adjusted for gender and age.

3 Confidence Intervals.

4 Estimates adjusted for gender and medical setting.

5 Estimates adjusted for age and medical setting.

6 Cytochrome P450 enzyme (CYP) 3A4.

The dispensed volumes of CYP3A4 dependent statins were similar among patients in the two groups of calcium channel blockers (mean DDD: 126, SD: 95) and (mean DDD 126, SD: 91) respectively. In contrast, the mean dispensed DDD (SD) for any statin was substantially higher in patients co-dispensed a fibrate 184 (151) as compared to patients on statin monotherapy 127 (93), (p = <0.001). The dispensed DDD (SD) for any statin in patients dispensed gemfibrozil and fenofibrate/bezafibrate was 178 (149) and 188 (150) respectively. This difference was not statistically significant (p = 0.056). The dispensed volumes of any statin in patients co-dispensed any of the three fibrates and in the subgroup of patients that were co-dispensed gemfibrozil in particular were also investigated in different subgroups. A similar marked increase in mean dispensed DDD was noted for primary care patients (175; SD 142 and 169; SD 142), for specialist care patients, (212; SD 168 and 214; SD 172) among patients 65 years or older (171; SD 138 and 159; SD 123) and in females (172; SD 145 and 162; SD 122).

## Discussion

In this population based study we showed that co-administration of statins with calcium channel blockers and fibrates respectively, associated with an increased risk of myopathy, may, to some extent, be avoided in clinical practice. We did not find evidence for avoiding concurrent prescriptions of statins and antibiotics with an increased risk of statin-induced myopathy. Co-prescribing of statins and fibrates, including gemfibrozil, is paradoxically associated with a marked increased statin dose, which further aggravates the risk for dose dependent adverse reactions.

The results should be considered in the context of several limitations. Due to the limited number of patients on statins and different airway antibiotics, the confidence interval around the adjusted odds ratio were actually quite large (adjusted OR in high dose and low dose patients 0.87; 95% CI 0.60–1.25 and 0.92; 95% CI 0.69–1.23). Hence the weak, however not statistically significant association measured, may very well be stronger. Even with similar estimates, the use of a larger study population, for example by using a longer study period including more users of antibiotics, could have turned the noted association significant. Although the data have the advantage of being based on information on dispensed rather than prescribed drugs, it was not possible to ascertain whether the medication was actually consumed. Furthermore, although we were able to adjust for gender and age, information regarding other potentially important confounders was missing. Thus, more detailed information on what the individual choice of lipid lowering therapy was based on such as indication for statin therapy (primary vs. secondary therapy), further cardiovascular risk stratifications (high risk vs. low risk), or presence of contraindications/intolerances, was missing. Another uncertainty about the dispensing data relates to the employment of a fixed time window to estimate the use of drug combinations. Although generally regarded valid, applying a time window may be associated with both under- or overestimation of exposure [42], [43]. The reason for the apparent similar prescription pattern of antibiotics in patients on different statins may, except for lack of knowledge regarding the potential risks of these specific drug-drug interactions, reflect a tendency to prioritize adherence to available guidelines for microbial usage. An alternative way to handle a potential interaction between a statin and a drug used for a shorter period of time may be to instruct the patient to suspend the statin prescription until the interacting prescription is complete. Unfortunately the present methodology using register based dispensing data is unable to evaluate such a strategy and may therefore to some extent have underestimated the clinicians’ ability to avoid this specific group of potential interactions. Furthermore, although excluding some other drugs of importance with respect to statin safety [2], the use of comparator drugs in the two different statins groups also enabled us to control for confounding by indication thus strengthening our conclusions. This approach relies on the assumption that *interacting* and corresponding *comparator drugs*, as well as the different statins, are used on similar/identical clinical indications, respectively. However, some differences may exist. Although the four calcium antagonists share the indications for hypertonia and angina pectoris, only verapamil and diltiazem can be prescribed for atrium fibrillation/flutter [35]. The possibility to avoid a potential interaction by switching to an alternative calcium channel blocker may therefore be limited; however, it still leaves the option open to use a statin whose metabolism is independent of CYP3A4.

The results from investigating the population as a whole were mostly consistent when focusing in the two investigated medical settings in the elderly and in females although some differences were noted. The decreased association between interacting statins and patients with verapamil/diltiazem was markedly more pronounced in patients treated in the specialist care as compared to patients treated in the primary care. This may indicate an increased knowledge concerning cardiovascular drug-drug interactions in the specialist care. Due to a decreased clearance resulting in an increased exposure for statins, elderly patients and females are more susceptible for drug interactions that may lead to myopathy [4], [8], [9]. Consequently this group of patients demands a pronounced care when prescribing medications. However, except for an unsignificant trend towards a decreased association between interacting statins and patients with erythromycin in the elderly no such care was noted. In fact, focusing on the females the significant decrease of interacting gemfibrozil in patients co-dispensed statins seen in the general population was lost (adjusted OR high dose and low dose statins 0.92; CI 0.68–1.24 and 0.90; CI 0.75–1.08).

Although there is quite an abundance of data regarding the crude prevalences of drug-drug interactions with a potential to cause myopathy in different contexts [44]–[48] information regarding to what degree clinicians actually avoid these interactions is limited. Ming et al. [44] and Bakhai et al. [45] performed two large nationally representative register based trials on the subject using US administrative/electronic medical records and General Practice Research Database respectively. Using the US administrative records, Ming et al. showed that the proportion of patients prescribed verapamil/diltiazem and macrolides was decreased in patients co-prescribed statins dependent on CYP3A4 for their metabolism as compared to non-3A4 statins (4.3% vs. 4.8% and 2.9% vs. 3.6%) which may indicate some degree of effort to avoid dangerous drug combinations on the behalf of the prescribing physician [44]. According to Bakhai et al. the proportion of verapamil/diltiazem in patients with atorvastatin and simvastatin was decreased as compared to in patients with rosuvastatin, pravastatin and fluvastatin (6.08% and 4.35% vs. 5.80%, 6.66% and 8.14% respectively), data that may point in the same direction [45]. Both studies also provided data on the exposures of a CYP3A4-metabolized statin with a labeled CYP3A4 inhibitor in patients 65 years or older. Thus, Ming et al. found that the prevalence of interactions was in the same level as compared to that of the population as a whole [44]. Bakhai and co-workers noted that for patients 65 years or older, the exposure for CYP3A4 inhibitors of any kind was similar in patients prescribed CYP3A4 statins vs. patients with non-3A4 statins, which resembled the picture seen in the population as a whole [45]. However, as this data was not stratified according to different inhibitors (i.e. calcium antagonists and macrolides) it was not possible to conclude on the age specific effort to avoid dangerous drug combinations in specific therapeutic groups. Devold et al. performed a large scale register study involving the entire Norwegian population that primarily focused on a new reimbursement policy favoring the use of simvastatin [48]. They noted that the reduction in use of statins without a potential to interact from 2004 to 2006 was similar in patients exposed and not exposed to CYP3A4 inhibitors which may indicate a lack of avoidance of statin associated drug interactions [48].

One alternative way of handling a pharmacokinetic interactions between two drugs used for a longer period of time may be to reduce the dose of the drug whose plasma exposure may be elevated. Interestingly, the statin dose in patients that were co-dispensed gemfibrozil was 40% higher than patients on statin monotherapy. This increase was similar when investigated in patients treated in primary care setting (+34%), even more pronounced in the specialist care setting (+69%), and also increased among patients 65 years or older (+25%) and in females (+28%). It is tempting to speculate that his may reflect a clash between safety regulations, that primarily focus on limiting the risk of adverse drug reactions, and clinical guidelines, emphasizing the importance to reach target lipid levels. Among patients that despite an increased statin dose do not reach these targets, a fibrate is added, a trend that may be more pronounced at the specialist care unit. Although this group of patients may very well be at higher risk for cardiovascular diseases, the decision to add gemfibrozil instead of bezafibrate or fenofibrate, without adjusting the statin dose, is unfortunate. Gemfibrozil interacts with statins not only pharmacodynamically but also increases its plasma level 1.5- to 3-fold which can result in plasma levels far exceeding the therapeutic range and an unacceptable benefit – risk balance [25]–[30].

In Sweden, SFINX, a software program used to warn for potential drug-drug interactions provide guidance on how to handle interactions, including the ones studied herein. It is available through either a website solution or as a part of a computerised decision support system (CDSS) that alerts the physician when about to prescribe a potentially inappropriate combination of drugs. This CDSS covers the prescriptions of approximately 80% of Swedish physicians [36], [49]. Apparently the Swedish physicians often fail to take advantage of this tool. Prescribers’ tendency to override DDI alerts is a well known problem described from several clinical contexts [50]–[53]. The present study further emphasizes the need to overcome this barrier.

In conclusion, prescribers may to some extent avoid co-prescription of statins with calcium blockers and fibrates with an increased risk of myopathy. We found no evidence for avoiding co-prescriptions of statins and antibiotics with an increased risk of statin-induced adverse drug reactions. Co-prescription of statins and gemfibrozil is paradoxically associated with a marked increased statin dose, further aggravating the risk for severe myopathy.
